# The effectiveness of a community-orientated model of primary care for type 2 diabetes compared to standard care

**DOI:** 10.4102/phcfm.v17i1.4912

**Published:** 2025-10-03

**Authors:** Shivani Pillay, Michael K. Pather

**Affiliations:** 1 Department of Family and Emergency Medicine, Faculty of Medicine and Health Sciences, Stellenbosch University, Cape Town, South Africa

**Keywords:** community-orientated primary care, effectiveness, diabetes, community, primary care

## Abstract

**Background:**

Non-communicable diseases constitute the primary cause of mortality in South Africa, surpassing infectious diseases. Among these, diabetes mellitus is the second leading cause of death. Although local literature on community-orientated primary care (COPC) remains limited, international evidence supports its effectiveness.

**Aim:**

This study aimed to evaluate the effectiveness of a COPC model for adults with type 2 diabetes compared to the standard facility model of care.

**Setting:**

The Chiawelo COPC (Chiawelo Community Practice) and Chiawelo Community Health Centre (CHC) in Soweto, South Africa.

**Methods:**

A cross-sectional observational study was conducted. File records of adults with type 2 diabetes from both models of care were examined for patients’ glycaemic control and clinicians’ adherence to evidence-based diabetes standards of care.

**Results:**

Chiawelo COPC (CCP) outperformed the CHC over the investigation period. Mean patient HbA1c at Chiawelo COPC (CCP) was lower than the CHC (7.9%, 8.9%; *p* < 0.001). Body mass index (BMI) (75%, 36%; *p* < 0.001), urine tests (74%, 42%; *p* < 0.001) and renal function assessments (95%, 80%; *p* < 0.05) showed superior results at the COPC practice. Differences were observed in examinations of feet (61%, 1%; *p* < 0.001) and eyes (38%, 1%; *p* < 0.001). Adherence discussions were more frequent at COPC (63%, 48%; *p* < 0.05).

**Conclusion:**

The COPC model is more effective than the standard facility-based model in managing type 2 diabetes in the Chiawelo community, Soweto.

**Contribution:**

This study contributes to understanding the effectiveness of a COPC model for diabetes care in a South African community.

## Introduction

Chronic non-communicable diseases (NCDs), including type 2 diabetes mellitus (T2DM), have emerged as leading contributors to the global burden of disease and continue to receive sustained attention within international development and health agendas. The World Diabetes Federation (WDF) estimated that in 2019, 463 million adults worldwide were living with diabetes, a figure projected to rise to 578 million by 2030 and 700 million by 2045 if comprehensive preventive and effective management strategies are not implemented.^1^ These projections highlight the urgency of adopting context-sensitive health system approaches to mitigate the escalating public health and economic impacts associated with diabetes. However, while the global burden provides important context, the necessity for focused local responses has become increasingly apparent, particularly in low- and middle-income countries (LMICs), such as South Africa, where diabetes is both a rapidly growing epidemic and a leading cause of mortality.^2^

In South Africa, the health system is confronted with a dual burden of disease, wherein NCDs have surpassed the impact of human immunodeficiency virus (HIV) and/or acquired immunodeficiency syndrome (AIDS) and tuberculosis in terms of mortality and healthcare utilisation.^2^ The South African Mortality and Causes of Death report (2018) placed diabetes as the second most common underlying cause of death over three years, reflecting a consistent upward trend in diabetes-related mortality.^2^ A cost-of-illness study estimated that diabetes in the public sector cost R2.7 billion and R21.8 billion in 2018 for diagnosed and undiagnosed patients, respectively.^3^ These costs are projected to escalate to R35.1 billion by 2030, with nearly half attributable to diabetes-related complications, underscoring the insufficiency of current care models to prevent downstream consequences of poor disease control.^3^

Current care models for diabetes management in South Africa are largely facility-based, fragmented and clinician-centric. People living with diabetes are typically required to attend routine follow-up appointments during working hours, creating access barriers that can contribute to non-adherence, loss to follow-up and poor clinical outcomes. Moreover, the overburdened public health system suffers from inadequate human resources, with a 2018 World Health Organization (WHO) report indicating that the African region’s health workforce density stood at 2.9 per 1000 population, well below the threshold of 13.4 needed to achieve universal health coverage targets.^4^ These systemic limitations necessitate a re-orientation of primary health care (PHC) services to focus on integrated, preventive and community-responsive care.

Community-orientated primary care (COPC) has been characterised as ‘the merger of front-line clinical medicine with public health’.^5,6^ In this way, COPC addresses the health requirements of individuals within the communal framework of family and community.^7^ The guiding principles of the COPC model are to encourage appropriate utilisation, which is supported through stakeholder involvement, re-orientated PHC services and population participation. The COPC model encompasses a PHC outreach team that includes a professional nurse, a health promotion officer and community health workers (CHWs). These PHC outreach teams are linked to the community practice in which the patients are seen.^8^ The COPC emphasises stakeholder involvement, re-engineered PHC services and population participation, implemented through ward-based outreach teams (WBOTs). These teams typically consist of a professional nurse, health promotion officer and CHWs, who collectively provide health promotion, screening, psychosocial support and follow-up services, including medication adherence support and home delivery of medication (HDM).^8,9^

During the COVID-19 pandemic, the reorganisation of primary care services in the Western Cape included a rapid rollout of HDM to people living with NCDs, including diabetes.^10^ Quantitative data analyses demonstrated improved glycaemic control among patients who received HDM compared to those who did not, while qualitative findings indicated strong patient preference for continued CHW-mediated services.^10^ These outcomes highlight the potential for COPC-aligned interventions to reduce the burden on facility-based services, improve clinical outcomes and align care delivery with the needs and preferences of patients.

Although the principles and theoretical underpinnings of COPC have gained broad support within South Africa, there remains a significant evidence gap regarding its effectiveness. A 2019 scoping review of COPC models in sub-Saharan Africa identified a need for greater emphasis on observational and experimental research to validate the effectiveness of COPC in diverse African contexts.^11^ Furthermore, an article in the *African Journal of Primary Health Care and Family Medicine* has echoed these calls for rigorous research, noting that while COPC is philosophically aligned with South Africa’s health reform goals, empirical evaluations within the region remain limited.^9^

The National Strategic Plan’s third and fourth goals – ensuring integrated, person-centred health services and promoting national capacity for high-quality research – further reinforce the need for evidence-based evaluations of COPC models tailored to local contexts.^12^ As such, research that assesses the effectiveness of COPC on chronic disease outcomes is both timely and necessary.

This study therefore seeks to address a critical gap in the literature by evaluating the effectiveness of the COPC model in managing type 2 diabetes within the South African context. Although international studies have demonstrated the broader benefits of COPC, there remains a dearth of data on the real-world effectiveness of this model in LMICs, particularly concerning measurable health outcomes. This gap is especially evident in South Africa, where resource constraints and the rising prevalence of diabetes have exposed the limitations of the traditional facility-based care model; however, there are no studies directly comparing it to COPC. By focusing on glycaemic control and adherence to treatment standards, this study aims to contribute context-specific evidence that can inform national health policy and models of care within the NHI framework while also contributing to the global discourse on effective, sustainable approaches to chronic disease.

### Aim

To evaluate the effectiveness of a community-orientated model of primary care for adults with type 2 diabetes compared to the standard facility model of care in the Chiawelo Community of Soweto, Gauteng.

### Objectives

- To assess the glycaemic control of adults with type 2 diabetes in both models of primary care.
- To assess the adherence of clinicians to diabetic clinical standards of care in both models of primary care.
- To compare the glycaemic control of adults with type 2 diabetes and adherence of clinicians to evidence-based diabetes clinical standards of care in a COPC practice and a community health centre (CHC).

### Outcome measures

#### Primary outcome measure

- Glycaemic control: Mean Glycosylated haemoglobin A1c (HbA1c)

Blood test: HbA1c refers to glycated haemoglobin, which identifies average plasma glucose concentration.

#### Secondary outcome measures

The following outcome measures assessed only if the screening guideline was followed and recorded in patient files. The outcome was not assessed:

- Screening for chronic conditions associated with diabetes: blood pressure (BP) checked and recorded at every visit.
- Cardiovascular risk assessment: BMI calculated and recorded annually.
- Cardiovascular risk assessment: Annual serum total cholesterol.
- Diabetic target organ damage screening: Annual urine examination to assess for proteinuria.
- Diabetic target organ damage screening: Annual foot examination done to assess for peripheral neuropathy, diabetic foot ulcers and signs of peripheral arterial disease.
- Diabetic target organ damage screening: Annual eye examination done to assess for signs of diabetic retinopathy (DR).
- Diabetic target organ damage screening: Annual serum creatinine and estimated glomerular filtration rates (eGFR) done to assess for diabetic nephropathy.
- Diabetic target organ damage screening: Annual serum potassium.
- Health promotion: Diet and exercise discussed.
- Health promotion: Adherence discussed.
- Health promotion: Influenza vaccine offered and administered.
- Health promotion: Smoking cessation discussed.

## Research methods and design

### Study design

A cross-sectional observational study using file reviews from both models of care to evaluate the glycaemic control and adherence of clinicians to evidence-based clinical standards of care in adults with type 2 diabetes.

### Setting

Chiawelo is a historically marginalised urban settlement within Soweto, established in 1956 as part of apartheid-era spatial planning to accommodate Xitsonga- and Tshivenda-speaking populations.^13^

The Chiawelo CHC is one of five key CHCs serving Soweto and is responsible for delivering health services to approximately 150 000 residents across five municipal wards.^13^ The facility comprises a network of prefabricated structures offering a comprehensive array of health services, including acute and chronic care, HIV and tuberculosis (TB) treatment, diagnostic imaging, a pharmacy, and 24-h emergency care. A variety of allied health disciplines, such as dentistry, occupational therapy, physiotherapy, speech therapy, and community psychiatry, are also represented. However, the delivery of care within the CHC remains largely fragmented, characterised by vertical programming where healthcare professionals often work in isolation across distinct service lines.^13^ Chronic disease management, particularly for conditions such as diabetes, is restricted to specific days. For instance, adult patients with diabetes are seen exclusively on Tuesdays and Wednesdays by PHC nurses stationed in the chronic care section. These patients typically arrive early in the morning and are seen on a first-come, first-served basis. In cases where medical input beyond nursing competencies is required, patients are referred to the acute outpatient department, necessitating a separate queuing process.

To address the limitations of this verticalised and reactive model, the Chiawelo Community Practice (CCP) was established in February 2014 on the premises of the CHC. Chiawelo Community Practice serves as a pilot initiative aligned with the objectives of South Africa’s National Health Insurance (NHI) and adopts a COPC approach. The model emphasises a shift from episodic, curative care to a more integrated and preventive orientation. Key operational drivers include active community engagement through CHWs, reorganisation of care delivery processes, intersectoral collaboration, and behaviourally-informed health promotion strategies.

The CCP team consists of a multidisciplinary team including a family physician, a clinical associate, a rotating cohort of family medicine registrars, medical interns on 2-week rotations, enrolled and professional nurses in team leadership roles, and 42 CHWs. This collective provides comprehensive care to over 30 000 individuals residing in Chiawelo.^13^ Regular weekly meetings are held between CHWs and clinical staff to discuss patient care challenges, share feedback, and conduct continuous in-service training. Additionally, every 5 weeks, collaborative quality improvement initiatives are undertaken by students and clinicians to strengthen adherence to clinical guidelines. Structured academic discussions occur biweekly, while quarterly stakeholder meetings foster community participation and intersectoral alignment.

As part of CCP’s foundational activities, CHWs conducted a full household enumeration in Ward 11, registering families for inclusion within the CCP system. This registration enables linkage to care and facilitates ongoing follow-up. The practice offers both acute and chronic services, including HIV management, and employs a digital appointment platform known as JoinaQ, which allows patients to book appointments via WhatsApp or through CHW referral. Upon arrival, patients are checked in using JoinaQ, either manually or through self-service.

Patient records are managed using a family-centred filing system, where all household members’ files are grouped together alongside a CHW file. This structure facilitates clinician-CHW collaboration during consultations and supports continuity of care in the community setting. Importantly, patients are managed in an integrated queuing system, regardless of their clinical condition or service needs. This contrasts with the fragmented care at the CHC, as patients with chronic conditions such as diabetes are not assigned to specific days or areas. CCP is currently expanding its digital infrastructure through a partnership with JoinaQ to implement a comprehensive electronic health record system. Although it was initially supported by private funding, CCP is now fully funded by the Gauteng Department of Health and operates under the budget of the Chiawelo CHC.

### Study population

The study population consisted of adults with type 2 diabetes receiving care at the Chiawelo Community Health Centre and the Chiawelo Community Practice.

### Inclusion criteria

- Adults with type 2 diabetes between the ages of 18 and 85 years.
- Adults with type 2 diabetes who have been on treatment for 1 year or more.
- Adults with type 2 diabetes who have been seen at their respective facility within the last year.

### Exclusion criteria

- Adults with diabetes who have not had an HbA1c done in the last 6 months.

### Sample size

In consultation with a biostatistician, the researcher estimated the mean HbA1c in the CHC group to be 8.8 and set out to detect a decrease of 7.8 in the COPC group. The assumed population standard deviation for each of the two groups was set at 2.2 based on existing literature. With a 90% power and a two-sided significance level (α) of 0.05, 77 adults with type 2 diabetes were required in each group. Based on these results, the researcher decided to use a sample size of 100 adults with type 2 diabetes in each group to account for any inaccuracies in the standard deviation that was assumed.

Although power calculations supported a sample size of 100 participants per group, practical and clinical considerations also guided this decision. This sample size enabled adequate representation of the local population with type 2 diabetes, capturing variability in disease profile, socioeconomic context and adherence patterns, while remaining feasible within the given resource constraints.

### Sampling

A systematic sampling technique was employed due to the absence of a register and the unknown population size of adults with type 2 diabetes at both the Chiawelo Community Practice and the Chiawelo CHC. At the Chiawelo Community Practice, files are stored in a designated file room, while at the Chiawelo CHC, files are kept in the chronic care department. From each site, every fifth file that met the inclusion criteria was systematically selected for review.

### Pilot study

A pilot study, using five individual files from each group, was conducted to test the data collection tool for ambiguity, ease of use, and to make overall improvements and changes where necessary on the quality of data collected.

### Data collection

A standardised data collection tool (Appendix 1) was developed based on the evidence-based South African National Department of Health standard treatment guidelines.^14^

This tool was used to extract key clinical data from patient files on glycaemic control and adherence to the diabetes clinical guidelines. The following variables were collected: HbA1c, blood pressure, and BMI done and recorded; annual serum total cholesterol, urine examination, foot and eye examination, serum creatinine with eGFR, and serum potassium; discussions on diet, exercise, adherence, and smoking cessation; influenza vaccine offered and administered.

Data were collected retrospectively using the REDCap mobile application by two trained final-year medical students from the University of the Witwatersrand, who were doing their Integrated Primary Care rotation at the Chiawelo CHC.

When a specific clinical test was not recorded in the patient file, it was assumed that the test had not been performed. Consequently, such cases were documented as ‘not done’ during data collection. This approach was consistent with the structure of the data collection tool, which framed each variable as a binary question, specifically asking whether the test was both conducted and recorded. Therefore, the absence of documentation was interpreted as a negative response, indicating non-compliance with the relevant clinical guideline.

### Data analysis

In conjunction with a biostatistician from the Epidemiology and Biostatistics department at Stellenbosch University, the data were analysed using the Statistical Package for the Social Sciences (SPSS) software. Descriptive statistics were computed to summarise the sample characteristics and the frequency of adherence to evidence-based diabetes clinical guidelines across the two models of care. Frequencies and percentages were calculated for binary variables such as blood pressure monitoring, BMI recording, urine dipstick testing, renal function tests, foot and eye examinations, dietary and adherence discussions, and preventive measures, including influenza vaccination and smoking cessation counselling.

For continuous data, specifically HbA1c levels, means and standard deviations were calculated. An independent samples *t*-test was conducted to compare the mean HbA1c values between the two facilities with a confidence interval of 95%. Equality of variances was assessed using Levene’s test, and the appropriate *t*-test results (with or without equal variances assumed) were reported. Effect sizes, including Cohen’s *d* and Hedges’ correction, were also calculated to quantify the magnitude of differences observed in glycaemic control. For categorical outcomes, cross-tabulations were performed to compare proportions between the two care models. Chi-square tests were used to assess the statistical significance of observed differences.

No imputation was conducted for missing data; instead, missing entries were interpreted as ‘not done’ per the data collection instrument, which required the presence of both the test and its documentation. As such, all analyses were performed on complete case data only. Outliers were not formally identified or excluded in this analysis. The decision to retain all data points, including potential outliers, was based on the study’s objective to evaluate routine clinical care as reflected in real-world medical records. Given the observational nature of the study and the retrospective data collection from facility records, all valid HbA1c values documented in patient files were included in the analysis, regardless of extremity, to preserve the ecological validity of the dataset. The range of HbA1c values (5.4% – 15.8%) fell within plausible clinical limits, and no extreme or biologically implausible values were observed that would warrant exclusion.

### Ethical considerations

The researcher applied for a waiver of consent as this retrospective collection of data posed minimal to no risk for the patients whose medical records were reviewed. No personal information was collected, and data were stored in password-protected files that are only accessible to the research team. Ethics approval was obtained from Stellenbosch University Health Research Ethics Committee (HREC reference number: S23/07/161). Permission was obtained from the Johannesburg district research committee to review folders from both facilities.

## Results

### Adherence of clinicians to diabetic clinical standards of care in both models of primary care

In the domain of blood pressure monitoring, both the Chiawelo COPC and Chiawelo CHC documented BP measurements during 99% of consultations (see Table 1). The statistical significance is affirmed by a *p*-value of 1.0, indicating the absence of a statistically significant difference between the two sites in this aspect.

**TABLE 1:** Comparison between Chiawelo community-orientated primary care and Chiawelo Community Health Centre in respect of adherence to evidence-based diabetes clinical standards of care.

Variable	COPC		CHC		*p*-value
	Count (*n*)	%	Count (*n*)	%	
**Was a BP done and recorded at every visit within the last year?**					1.000
Yes	99	99.0	99	99.0	-
No	1	1.0	1	1.0	-
**Was the BMI calculated and recorded in the last year?**					< 0.001
Yes	75	75.0	36	36.0	-
No	25	25.0	64	64.0	-
**Has a urine dipstick been done and recorded in the last year?**					< 0.001
Yes	74	74.0	42	42.0	-
No	26	26.0	58	58.0	-
**Has a serum creatinine and eGFR been done and recorded in the last year?**					0.001
Yes	95	95.0	80	80.0	-
No	5	5.0	20	20.0	-
**Has a serum potassium been done and recorded in the last year, if on an ACE-inhibitor or eGFR < 30 mL/min?**					0.346
Yes	36	36.0	27	27.0	-
No	16	16.0	21	21.0	-
N/A	48	48.0	52	52.0	-
**Has a serum total cholesterol been done and recorded in the last year?**					0.079
Yes	89	89.0	80	80.0	-
No	11	11.0	20	20.0	-
**Has a foot examination been done and recorded in the last year?**					< 0.001
Yes	61	61.0	1	1.0	-
No	39	39.0	99	99.0	-
**Has an eye examination been done and recorded in the last year?**					< 0.001
Yes	38	38.0	0	0.0	-
No	62	62.0	100	100.0	-
**Has diet education been discussed and recorded in the last year?**					0.350
Yes	74	74.0	68	68.0	-
No	26	26.0	32	32.0	-
**Has adherence been discussed and recorded in the last year?**					0.033
Yes	63	63.0	48	48.0	-
No	37	37.0	52	52.0	-
**Has the influenza vaccine been discussed and offered in the last year?**					0.174
Yes	4	4.0	1	1.0	-
No	96	96.0	99	99.0	-
**Has the influenza vaccine been administered in the last year?**					0.081
Yes	0	0.0	0	0.0	-
No	3	3.0	0	0.0	-
Not Available	97	97.0	100	100.0	-
**Has smoking cessation been discussed and recorded in the last year?**					0.006
Yes	7	7.0	0	0.0	-
No	4	4.0	11	11.0	-
N/A	89	89.0	89	89.0	-

BMI, body mass index; eGFR, estimated glomerular filtration rates; BP, blood pressure.

Patient education and examination of serum potassium yielded *p*-values of 0.350 and 0.346, respectively, indicating no statistically significant difference between the two sites. Nevertheless, discussions on adherence were more prevalent at the Chiawelo COPC (63%) than at the Chiawelo CHC (48%), with a *p*-value of 0.033.

There was a marginal difference in serum cholesterol tests conducted, with an adherence of 89% in the Chiawelo COPC compared to the Chiawelo CHC’s 80%. The non-statistically significant nature of this difference is shown by a *p*-value of 0.079.

In terms of influenza vaccination, both sites exhibited limited engagement in discussions and administration. Clinicians at the COPC discussed the vaccine with 4% of patients and administered it to. In comparison, the CHC discussed the vaccine with 1% of patients, with no statistically significant difference (*p*-values of 0.174 and 0.081, respectively).

A marked disparity, between the COPC and the CHC, is evident in the calculation and documentation of BMI (75% and 36%, respectively), administration of urine dipstick analysis (74% and 42%, respectively), and the evaluation of renal function using serum creatinine and eGFR calculations (95% and 80%, respectively). The statistical significance of this disparity is confirmed by a *p*-value of 0.001 for all three variables.

The most notable differences are observed in the percentage of foot and eye examinations. Foot examinations were conducted in 61% of patients from the COPC, in contrast to only 1% at the CHC, while eye examinations were performed in 38% of files reviewed from the COPC compared to 0% at the CHC. These results yield *p*-values below 0.001.

### Glycaemic control of adults with type 2 diabetes in both models of primary care

At the Chiawelo COPC, the mean HbA1c was 7.9 (see Table 2), in contrast to the Chiawelo CHC, where the mean is significantly higher at 8.9. The difference between the two is deemed statistically significant as indicated by a *p*-value of less than 0.001.

**TABLE 2:** Comparative glycaemic control of adults with type 2 diabetes at the Chiawelo community-orientated primary care and Chiawelo Community Health Centre.

Variable	Chiawelo COPC	Chiawelo CHC	*p*-value
**What is the measurement of the HbA1c done within the last 6 months?**			< 0.001
Mean	7.9	8.9	-
Standard deviation	1.7	1.6	-
Minimum	5.4	7.0	-
Maximum	13.0	15.8	-

COPC, community-orientated primary care; CHC, Community Health Centre.

### Key findings

This research study presents an evaluation of the effectiveness of a COPC model for adults with type 2 diabetes, compared to the standard facility-based model of care in Chiawelo, Soweto. The clinical parameters evaluated included HbA1c levels, BP monitoring, BMI calculations, urine dipstick tests, serum creatinine and eGFR measurements, serum potassium levels, total cholesterol testing, and the fulfillment of preventive assessments such as foot and eye examinations. Moreover, the frequency of dietary education, adherence discussions, influenza vaccinations and smoking cessation counselling was assessed.

The data suggest a marked difference in effectiveness between the two models of care, with the Chiawelo COPC consistently exceeding the Chiawelo CHC across most parameters. The mean HbA1c level at Chiawelo COPC was substantially lower (7.9%) in comparison to Chiawelo CHC (8.9%), with a *p*-value below 0.001, indicating enhanced glucose regulation at Chiawelo COPC. The Chiawelo COPC also demonstrated superior outcomes in BMI calculations (75% vs. 36%), urine dipstick tests (74% vs. 42%), and renal function evaluations (95% vs. 80%), all displaying statistically significant variances (*p* < 0.001 for BMI and urine dipstick, *p* = 0.001 for renal function tests).

Preventive care indicators additionally emphasised significant disparities, as the Chiawelo COPC conducted foot examinations in 61% of individuals with type 2 diabetes compared to 1% at the Chiawelo CHC, and eye examinations in 38% of patients compared to none at the CHC, both with *p*-values below 0.001. Conversations regarding adherence were more prevalent at COPC (63% vs. 48%, *p* = 0.033), while both the COPC and the CHC exhibited limited involvement in influenza vaccination and smoking cessation counselling. Both the Chiawelo COPC and the CHC adhere rigorously to the established standard treatment guidelines for blood pressure recording, with no notable difference between the two (99% vs. 99%).

## Discussion

### The glycaemic control of adults with type 2 diabetes in both models of primary care

The substantial difference in HbA1c between the Chiawelo COPC and Chiawelo CHC highlights the effectiveness of adherence to comprehensive diabetes care protocols. The decreased mean HbA1c at COPC signifies superior glycaemic control, a critical aspect in averting long-term microvascular and macrovascular complications.^15^

### Adherence of clinicians to diabetic clinical standards of care in both models of primary care

A causal relationship has been demonstrated between elevated BMI levels and a heightened susceptibility to Diabetic Nephropathy (DN) as well as reduced estimated glomerular filtration rates (eGFR).^16^ Additionally, it was found that the escalation in BMI levels had a more pronounced effect on the risk of DN in the female population.^16^ Therefore, calculating, recording, and monitoring the BMI of individuals with type 2 diabetes remains paramount in an attempt to prevent complications. Resource distribution and adequate staffing continue to be a challenge in the PHC setting. These constraints may cause a prioritisation of acute healthcare services over preventative interventions, leading to non-adherence to recommended clinical guidelines. The COPC approach to primary care focuses on shifting the focus of health services from curative to preventative, and the Chiawelo COPC has re-orientated its services to reflect this. Community health workers received training in calculating BMI and are now utilised at the COPC and within the broader community for this purpose.

Current diabetes guidelines focus on preventive care measures to avert diabetes complications. Preventive foot examinations for people living with diabetes are linked to a reduction in hospitalisations due to diabetes-related complications.^17^ Given the critical role of foot and eye examinations in preventing diabetes complications, the extremely low rate of these screening examinations (1% and 0%, respectively) by the Chiawelo CHC in comparison to the Chiawelo COPC is concerning. The Chiawelo CHC operates within a traditional organisational structure where chronic disease management is mainly the responsibility of the nurse. This poses a significant challenge as a study conducted in the Sol-Plaatje district in the Northern Cape revealed that out of the 128 professional, auxiliary, and enrolled nurses, only 58.1% knew that South African Diabetic Foot Guidelines existed, while 57.7% had read them. Approximately 29.8% had never attended a class on diabetic foot care, and 85.6% required training on diabetic foot care.^18^

The Society for Endocrinology, Metabolism and Diabetes of South Africa (SEMDSA) recommends that patients should be screened for DR by an ophthalmologist or an optometrist who is trained in detecting DR.^19^ However, in limited resource settings, this is not realistic. Despite earlier controversies regarding the reliability of visual acuity examinations for detecting DR, current guidelines emphasise its significance in settings with limited resources, particularly when ophthalmologists are not available to conduct screenings.^20^ The involvement of PHC nurses in DR screening demonstrates variability across different provinces. It was established that PHC nurses in KwaZulu-Natal performed pinhole testing (57.1%), external eye examination (85.7%), and visual acuity testing (76.2%).^21^ Conversely, their counterparts in Limpopo only conducted visual acuity examinations (40%).^22^

The organisational culture at the Chiawelo COPC promotes a team-based approach to care, fostering collaboration among all involved healthcare providers and ensuring coordinated care for patients. Many of the functions and skills related to the COPC framework extend beyond the realm of clinical practice. Nevertheless, this does not diminish the importance of ensuring the delivery of quality clinical services.^23^ The Chiawelo COPC team consists of CHWs led by a nurse and supported by clinical associates, interns, and family physicians who deal with both acute and chronic care. This team composition enables the administration of advanced screening examinations, such as fundoscopy, to all individuals with type 2 diabetes who attend the COPC.

Patient education regarding the management of diabetes, encompassing the significance of regular HbA1c monitoring, control of BMI, and lifestyle adjustments, plays a pivotal role in enabling patients to engage actively with their health. This objective is achieved utilising a dual process at the Chiawelo COPC, through clinicians during patient consultations and targeted health promotion by CHWs in the community. Healthcare providers at the Chiawelo CHC are often inundated with patients, leaving them with limited time for health promotion during the consultation.

Diabetic nephropathy (DN) represents a significant microvascular complication associated with diabetes mellitus.^24^ At present, diabetic nephropathy is the predominant cause of end-stage renal disease (ESRD).^24^ Annual urine dipstick and serum creatinine with eGFR form part of the national standard treatment guidelines^14^ to monitor renal function in individuals with type 2 diabetes. It is essential to recognise that designated personnel are responsible for collecting blood samples at both facilities. Therefore, additional research is necessary to evaluate the inadequate implementation of clinical guidelines at the Chiawelo CHC.

### Clinical outcomes with no statistical significance

Some variables in this study yielded non-significant *p*-values, including serum cholesterol testing, influenza vaccination, and smoking cessation counselling. While the lack of statistical significance suggests that there is no meaningful difference between the COPC and CHC models for these specific indicators, it does not diminish the clinical relevance of these practices. Instead, these findings likely reflect broader systemic factors that influence the delivery and documentation of preventive care within both models.

The absence of a significant difference in serum cholesterol testing indicates that lipid monitoring is a routinely implemented component of diabetes care across both sites, possibly due to adherence to national clinical protocols.

In the population of working-age individuals diagnosed with diabetes, the administration of influenza vaccination was effective in reducing hospitalisations. However, it did not impact mortality outcomes.^25^ Concerning elderly diabetic individuals, a meta-analysis revealed that there is a level of protection against all-cause mortality as well as hospitalisations.^25^ The low level of engagement with influenza vaccines and smoking cessation counselling at both sites indicates a notable missed opportunity in preventive care.^26^ It is noteworthy to mention that influenza vaccines were not accessible at either site during the year in which this study was conducted and the preceding year. Therefore, the low engagement with influenza vaccination across both models is attributed to structural barriers.

In addition, the minimal incorporation of smoking cessation counselling may reflect systemic constraints, including competing clinical priorities, limited time during consultations, a lack of structured cessation programmes or insufficient provider training in behaviour change counselling.

### Comparison to similar models of care

Research on COPC is currently inadequate.^9,11,27^ With no studies focusing on the effectiveness of COPC on diabetes or other chronic conditions, however, the findings from this study are consistent with existing literature that has evaluated similar care models.

Population-based care involves the utilisation of guidelines, epidemiologic data, and techniques to strategise, coordinate, deliver and monitor care for specific clinical sub-populations such as individuals with diabetes.^28^ Population-based care shares common characteristics with the COPC model, particularly in its comprehensive approach to healthcare delivery aimed at improving the health outcomes of entire populations. Both models emphasise a preventive approach and are data-driven, utilising epidemiological data and health indicators to identify health trends and disparities, thereby informing targeted interventions. An evaluation of a multifaceted programme of support on the ability of primary care teams to deliver population-based diabetes care in Washington, United States, was conducted between 1993 and 1996.^29^ After the implementation of the programme, HbA1c testing increased from 77% in 1994 to 80% in 1996. Similarly, retinal screening rates improved from 46% in 1993 to nearly two-thirds of diabetic patients by 1996. Prior to 1996, less than 20% of patients received foot care. However, within a year, 50% of patients had foot examinations recorded.^29^

The chronic care model (CCM) is an evidence-based framework for organising and optimising diabetes care delivery by modifying essential healthcare system elements to facilitate high-quality patient-centred management.^30,31^ These six elements are the organisation of the healthcare delivery system, community linkages or resources, self-management support, decision support, delivery system design, and clinical information systems, and have been used as interventions to demonstrate improvement in diabetes care.^31,32^ A systematic review and meta-analysis were conducted to evaluate the effectiveness of the CCM for adults with type 2 diabetes in primary care.^33^ Meta-analysis of the 17 randomised controlled trials (RCTs) revealed that CCM interventions significantly decreased HbA1c levels compared to usual care with a mean difference (MD) of –0.21%, *p* < 0.00001.^33^

Team-based care (TBC), to improve diabetes management, is a health systems-level, organisational intervention that incorporates a multidisciplinary team to help patients manage their diabetes. Each team includes the patient, the patient’s primary care provider (not necessarily a physician), and one or more other health professionals. The TBC was developed to pursue the ‘Triple Aim’ of reducing cost, improving health outcomes and enhancing patient experience.^34^ The TBC model shares similarities with the COPC care model as they both incorporate a multidisciplinary team strategy. A meta-analysis was conducted to evaluate the effectiveness of TBC in managing diabetes. A random effect meta-analysis of the 35 RCTs identified, revealed that TBC, compared to controls, was associated with more significant reductions in blood glucose levels (–0.5% in HbA1c) and greater reductions in blood pressure and lipid levels.^35^ Furthermore, the interventions resulted in an increased percentage of patients achieving target levels for blood glucose, blood pressure and lipids, as indicated by the guidelines of the American Diabetes Association that were accessible at that time.^35^

### Implementation of COPC: Contextual considerations

The implementation of COPC varies substantially across rural and urban settings in South Africa. Rural areas face pronounced challenges, including shortages of healthcare professionals, logistical barriers to outreach, limited infrastructure, and dispersed populations that hinder service delivery and community engagement.^36,37^ Although some rural municipalities have achieved success through strategic partnerships and the involvement of traditional leadership, these remain exceptions.^37^ Conversely, urban areas, while better resourced and equipped with stronger infrastructure and health networks, must contend with high population mobility, complex social dynamics, and increased service demand.^38^ These contrasting contexts necessitate tailored approaches to COPC implementation.^8^

Socioeconomic determinants critically shape both the delivery and outcomes of COPC. Structural inequalities – such as poverty, low educational attainment, and limited access to services – affect community participation and impact the model’s effectiveness and sustainability.^7,36,37^ The Chiawelo model’s success may not readily translate to settings with more severe social vulnerabilities without appropriate contextual adaptations.

Resource allocation and system integration also play a decisive role. The Chiawelo COPC model was implemented within a specific resource environment, which may not be replicable elsewhere without adjusting for regional disparities in funding, infrastructure, and institutional capacity.^37,38^ Similarly, the availability and distribution of skilled personnel are pivotal to the effective delivery of COPC. Human resource constraints, especially in rural regions, pose significant barriers.^36^ Therefore, flexible team configurations and targeted capacity-building are essential to scale COPC equitably and effectively across diverse healthcare settings.

### Strengths and limitations

This research study used an exhaustive and comparative study design to evaluate the effectiveness of two distinct primary care models. Through a systematic analysis encompassing various clinical indicators, a comprehensive perspective on the quality of diabetes management is presented for both sites. This diverse set of data facilitates a nuanced comprehension of how diverse care models influence clinical outcomes.

The Chiawelo Community Practice offers valuable insights into the potential of the COPC model to address healthcare challenges in South Africa. However, its implementation in a specific peri-urban environment with particular infrastructural and resource characteristics limits the direct transferability of findings to other South African healthcare contexts. Multiple contextual factors, including geographical location, socioeconomic conditions, resource availability, and cultural considerations, influence the effectiveness of COPC implementation. As a result, the outcomes might not accurately reflect the national or global circumstances.

It is essential to recognise that the study employed a cross-sectional design, which inherently limits the capacity to establish causal relationships between the implementation of COPC and improvements in diabetes-related outcomes. As an observational study, the findings demonstrate associations between the COPC model and selected diabetes management indicators; however, they do not provide definitive evidence of a causal link.

The researcher had to depend on pre-existing medical records; hence, the accuracy and comprehensiveness of data hinged on the diligence and precision of record-keeping practices at both facilities. A potential limitation of the data collection approach is that it assumes the absence of documentation equates to the absence of clinical action. By coding missing entries as ‘not done’, there is a risk of underestimating the actual delivery of care, particularly in resource-constrained settings where documentation practices may be inconsistent or incomplete. This method may conflate poor record-keeping with non-performance of clinical tasks, thereby misrepresenting the true level of adherence to clinical guidelines. While this conservative approach ensures standardisation and objectivity in data analysis, it may bias the findings towards lower performance indicators, especially in facilities where documentation is not rigorously enforced.

This study excluded folders of individuals whose HbA1c levels had not been taken within the last 6 months. While this exclusion was necessary to evaluate the primary outcome of glycaemic control, it may have inadvertently excluded cases where adherence to other guideline-based standards of care (e.g., BMI calculation, foot examinations, urine dipstick tests) was also poor. This could have skewed the findings related to secondary outcomes, as the sample may have been biased towards cases with better overall care and adherence.

An additional limitation of the study is its emphasis on clinical metrics, without incorporating direct assessments of patient-centred outcomes such as quality of life, patient satisfaction, or adherence to treatment from the patient’s perspective.

### Implications for practice and further research

This study highlights the critical need to shift PHC services to utilise a COPC model to enhance the management of type 2 diabetes. The COPC approach, with its emphasis on prevention, patient-centred care and the integration of CHWs, has demonstrated improved glycaemic control and adherence to clinical standards compared to the standard facility-based model. These findings suggest that implementing COPC at the facility level and not just in the community can address the gaps in diabetes care by providing comprehensive, continuous and coordinated care.

The COPC model has shown effectiveness in managing type 2 diabetes within the Chiawelo community; however, scaling this approach across the broader South African PHC system, particularly within the context of the NHI framework, presents both opportunities and challenges. Key barriers to scaling include resource constraints, limited health system capacity, integration difficulties for CHWs, inadequate data management and health information systems, and the need for meaningful community engagement. Overcoming these obstacles will require substantial investment in CHW training, reinforced PHC infrastructure, clearly delineated CHW roles, interoperable information systems and culturally responsive engagement strategies.

Conversely, several facilitators support the expansion of COPC, including the NHI’s focus on PHC, an established national policy framework for CHWs, existing evidence of COPC’s effectiveness, its alignment with principles of task-shifting and decentralisation, and opportunities for collaboration between government, healthcare providers, non-governmental organisations and communities. By strategically addressing implementation barriers while leveraging these facilitators, South Africa can effectively scale the COPC model under the NHI, enhancing access to high-quality PHC and alleviating the burden of chronic diseases.

Adapting the COPC model for different South African settings requires careful consideration of contextual factors and the development of flexible implementation approaches. Rather than viewing the Chiawelo model as a rigid template, it should be seen as a source of principles and practices that can be thoughtfully adapted to diverse healthcare environments. Future research should focus on implementing and evaluating COPC across a range of South African healthcare settings, specifically examining how the model can be effectively adapted to different contexts while maintaining its core principles. Such research would contribute to the development of more robust, context-sensitive approaches to PHC that can better address South Africa’s complex health challenges.

To more rigorously evaluate the long-term impact of COPC on diabetes management, future research should adopt longitudinal designs, such as RCTs or prospective cohort studies. These methodologies would facilitate the tracking of patient outcomes over time and provide more robust evidence regarding the sustained effects of COPC on clinical indicators. In addition, future studies should control for potential confounding variables, such as socioeconomic status, access to healthcare, and comorbidities, to yield a more comprehensive understanding of the relationship between COPC implementation and diabetes-related outcomes.

Further research should also explore the effectiveness of COPC in managing other NCDs to assess its broader applicability and benefits. Importantly, forthcoming studies should integrate patient-centred outcomes, including quality of life and patient satisfaction, to ensure a more holistic evaluation of the COPC model.

### Recommendations

Based on the findings of this study, it is recommended that the implementation of the COPC model in PHC facilities be prioritised. The PHC system in South Africa is organised around a commitment to COPC, which is seen practically through the widespread implementation of WBOTs.^39^ Despite this, progress to shift service delivery in the facilities, to a more holistic approach using the COPC model, has been slow, with WBOTs usually working in the community in a silo.

District managers should prioritise restructuring CHCs to represent a COPC model and integrate WBOTs into the care team at the facility. Regular monitoring and evaluation of diabetes care practices using clinical audits and quality improvement cycles should be implemented to ensure adherence to evidence-based clinical guideline recommendations and to identify areas for improvement.

## Conclusion

The COPC model has demonstrated superior effectiveness in managing type 2 diabetes compared to the standard facility-based model in the Chiawelo community of Soweto. This study found that the COPC model consistently yielded better outcomes across several clinical parameters, including glycaemic control, adherence to diabetes care standards, and preventive screenings. Adults with type 2 diabetes from the Chiawelo COPC practice had notably lower HbA1c levels (7.9%) compared to those in the Chiawelo CHC (8.9%), with a statistically significant difference (*p* < 0.001). By focusing on prevention, patient-centred care, and leveraging the role of CHWs, the COPC model addresses key gaps in diabetes management, leading to better health indicators. The findings underscore the importance of reorienting PHC services towards a more integrated and collaborative approach. Implementing COPC can significantly enhance the quality of care for patients with chronic diseases, contributing to the achievement of national and global health goals. Further research is essential to evaluate the effectiveness of the COPC model on other health outcomes.

## Figures and Tables

**FIGURE 1:**
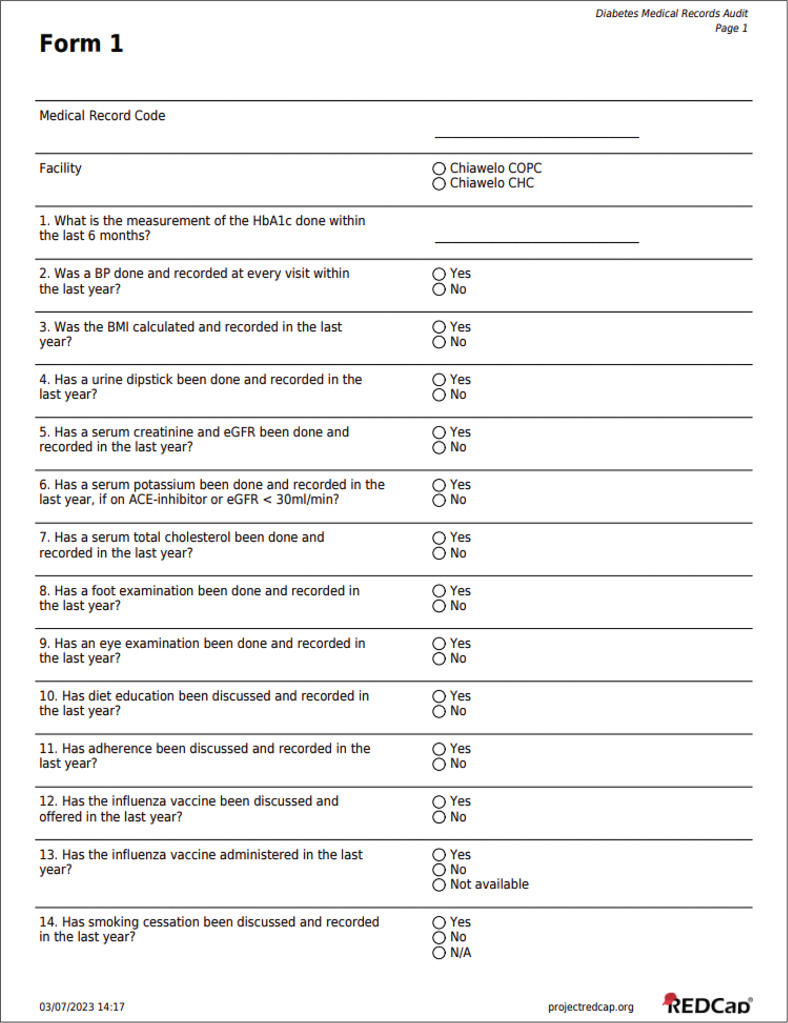
Data collection tool.
